# Virulence Gene Expression of *Staphylococcus aureus* in Human Skin

**DOI:** 10.3389/fmicb.2021.692023

**Published:** 2021-06-11

**Authors:** Ana Rita Cruz, Jos A. G. van Strijp, Fabio Bagnoli, Andrea G. O. Manetti

**Affiliations:** ^1^GSK, Siena, Italy; ^2^Department of Medical Microbiology, University Medical Center Utrecht, Utrecht University, Utrecht, Netherlands

**Keywords:** *Staphylococcus aureus*, human skin, virulence factors, gene expression, pathogenicity, niche, sweat glands

## Abstract

*Staphylococcus aureus* is the main cause of human skin and soft tissue infections. However, *S. aureus* pathogenicity within the skin is not fully characterized. Here, we implemented an *S. aureus* cutaneous infection model using human skin explants and performed a time-course infection to study the gene expression profile of a large panel of virulence-related factors of *S. aureus* USA300 LAC strain, by high-throughput RT-PCR. We pinpointed the genes that were differentially regulated by the bacteria in the skin tissues and identified 12 virulence factors that were upregulated at all time points assessed. Finally, using confocal microscopy, we show that the expression of alpha-hemolysin by *S. aureus* varies dependent on the skin niche and that the bacteria preferentially accumulates inside sweat glands and ducts. Taken together, our study gives insights about the pathogenic lifestyle of *S. aureus* within human skin tissues, which may contribute for the development of anti-*S. aureus* therapeutic strategies.

## Introduction

*Staphylococcus aureus* is an important human pathogen that persistently colonizes 20% of human population and transiently colonizes another 60% (Kluytmans et al., 1997). These bacteria can cause different diseases that range from skin and soft tissue infections (SSTIs) as impetigo, folliculitis, abscesses and cellulitis (Olaniyi et al., 2016), to more invasive diseases like endocarditis, osteomyelitis, pneumonia and sepsis (Lowy, 1998; Salgado-Pabón and Schlievert, 2014). Available antibiotics are not sufficiently effective against multidrug resistant *S. aureus* strains and therefore this pathogen represents a major concern for human health. Although several *S. aureus* vaccines were tested in clinical trials, they failed to show efficacy in humans (Bagnoli et al., 2012). The overreliance on animal models and the incomplete understanding of *S. aureus* pathogenesis during human infection may explain this failure.

*S. aureus* is equipped with a large array of virulence factors that include adhesins, immune evasion factors, toxins and proteases (Foster, 2005; Foster et al., 2014; Thammavongsa et al., 2015; Tam and Torres, 2019). Complex regulatory networks modulate the expression of metabolic and virulence factors and enable the bacteria to adapt to different host environments (Cheung et al., 2004; Balasubramanian et al., 2017; Haag and Bagnoli, 2017; Jenul and Horswill, 2018). A number of global regulators are influenced by environmental stimuli as nutrients and oxygen availability, cell density, pH and osmolarity (Cheung et al., 2004; Balasubramanian et al., 2017; Haag and Bagnoli, 2017; Jenul and Horswill, 2018). Moreover, there is increasing evidence that host niche-specific factors also have an impact on *S. aureus* gene expression (Rothfork et al., 2003; Cheung et al., 2004). The best well-characterized regulator of *S. aureus* virulence is the accessory gene regulator (agr), a quorum-sensing system. Generally, *S. aureus* expresses virulence factors that facilitate adhesion to the host tissues as surface-bound proteins in the beginning of infection (or mid-exponential phase), while an activated agr system leads to the expression of secreted toxins and proteases that enable tissue invasion and dissemination later in infection (or late-exponential/stationary phase) (Cheung et al., 2004; Le and Otto, 2015).

The skin tissues are often the primary site of *S. aureus* infection, from which bacteria can spread to other organs to cause more invasive diseases. However, little is known about *S. aureus* pathogenicity during infection of human skin tissues. Because human skin differs from mouse skin in both histology and immunology (Pasparakis et al., 2014) and *S. aureus* produces human-specific virulence factors, it is important to study *S. aureus* infections in human tissues. To our knowledge, only two studies analyzed the transcriptional response of *S. aureus* in human skin (Loughman et al., 2009; Date et al., 2014). These studies were performed in clinical samples of cutaneous abscesses, which, although relevant, do not permit to investigate gene expression of *S. aureus* at initial stages of infection and also limit the comprehension about transcription kinetics while infection evolves.

In this study, we assess the virulence gene expression of *S. aureus* USA300 LAC strain in human skin tissues over time and highlight the factors that might be required to establish and/or maintain *S. aureus* cutaneous infections. Additionally, we show that *S. aureus* accumulates in sweat glands and demonstrate that the transcription profile of the bacteria also varies within skin niches. Overall, our study contributes for a better understanding of *S. aureus* pathogenicity on SSTI progression.

## Materials and Methods

### Ethics Statement

The studies involving human participants were reviewed and approved by French Ministry of Higher Education, Research and Innovation. The patients/participants provided their written informed consent to participate in this study.

### Bacterial Strains and Culture

*S. aureus* USA300 LAC strains were grown overnight in tryptic soy broth (TSB) at 37°C, diluted 1:100 in fresh TSB and cultured at 37°C until mid-log phase (OD_600_ 0.6–0.7). Cells were centrifuged, washed and resuspended in PBS to obtain 5 × 10^6^ CFU/μL. For USA300 LAC pOS-hla-mCherry, the promoter of *hla* gene was fused to *mCherry* in a pOS plasmid backbone and transformed into USA300 LAC strain.

### Process and Infect Fresh Human Skin Explants

**FIGURE 1:**
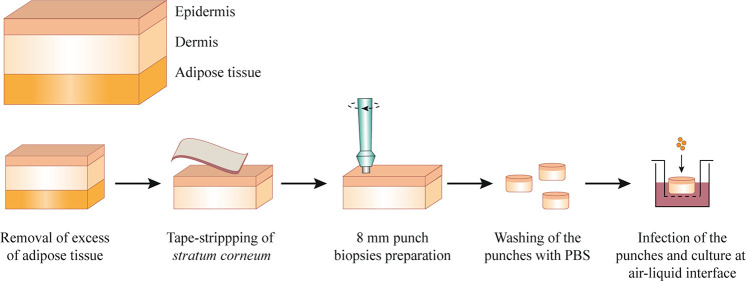
Schematic representation of the protocol to process and infect fresh human skin explants. After removal of the adipose tissue, the skin explants were pinned in a dissection board and the surface of epidermis was tape-stripped with an hypoallergenic tape. Skin punches of 8 mm were prepared, washed in PBS and placed in a transwell, previously filled with culture medium. The skin was infected with 5 × 10^6^ CFU of USA300 LAC strain and cultured at air-liquid interface for 2, 3, 24, or 72 h.

Human skin explants were processed as described before (Olaniyi et al., 2018), with modifications (Figure 1). In short, after removal of adipose tissue, the skin sheet was stripped 30 times with a hypoallergenic tape (Transpore, 3M). Using disposable biopsy punches (Kai Medical), 8 mm biopsies in diameter were collected and subsequently submerged in culture medium (Advanced DMEM/F-12 supplemented with 4 mM L-Glutamine; Gibco). After two washes with PBS, the punches were placed in 12-well transwell plates with 0.4 μm pore size (Corning), previously filled with 1 mL of culture medium. The transwells enable culture of the skin at air-liquid interface (the surface of the epidermis layer is exposed to the air, while the other skin layers are submerged in the culture medium), as depicted in Figure 1. Finally, the punches were infected with 5 × 10^6^ CFU USA300 LAC. To ensure that the bacteria does not contact with the culture medium, we carefully placed 1 μL of bacteria on top of the dry epidermis. After infection, the skin was cultured at air-liquid interface for 2, 3, 24, or 72 h, at 37°C, 5% CO_2_. For the early time points (2 and 3 h of infection), non-adherent bacteria were removed by washing the skin surface twice with PBS before RNA isolation. This protocol was performed using skin explants collected from three different donors and technical duplicates or triplicates were performed for each time point.

### Confocal Microscopy

Skin punches were fixed with 4% formaldehyde, overnight at RT. The punches were washed in PBS, embedded in tissue freezing medium (Leica Biosystems), snap-frozen in a dry ice-ethanol bath and stored at –80°C. Leica cryostat CM1950 was used to cut 8 μm-thick cryosections that were mounted onto SuperFrost Plus adhesion slides (Thermo Fisher Scientific). Cryosections were permeabilized with staining buffer (0.3% Triton-100, 3% BSA in PBS) for 1h at RT, in a humidity chamber, and stained with antibodies. The following antibodies were used: rabbit anti-wide spectrum cytokeratin (Abcam), rabbit anti-cleaved caspase-3 (Abcam), anti-*Staphylococcus aureus* antibody biotin (Abcam), Alexa Fluor 647 Phalloidin (Life technologies), goat anti-rabbit Alexa Fluor 568 (Life Technologies) and streptavidin Alexa Fluor 488 (Life technologies). Primary antibodies were incubated for 1h at RT and, after three washes with PBS, secondary antibodies were added for 30 min at RT, in the dark. After antibody staining, slides were mounted with ProLong Gold Antifade Reagent with DAPI (Life Technologies). Confocal images were acquired using Zeiss LSM 700 confocal microscope.

### Two-Photon Microscopy

For two-photon microscopy, skin punches were infected with a GFP-expressing USA300 LAC strain for 72 h. The punches were fixed with 4% formaldehyde overnight at RT, washed in PBS and stored at –80°C until imaging.

### RNA Extraction From Infected Skin Tissues, cDNA Synthesis and qPCR

Infected skin punches were incubated in RNAlater (Thermo Fisher Scientific) up to 1 week, at 4°C. Tissue disruption was performed in cold TRIzol (Invitrogen) using a Tissue Homogenizer (Omni International) and sterile OMNI Hard Tissue Tips (Omni International). The skin punches were submitted to 3 cycles of disruption of 60 s at maximum speed and incubated on ice for 2 min in between each run. After that, the samples were transferred to Lysis Matrix B tubes (Mp Biomedical) and 2 cycles of lysis of 60 s at 6.5 m s^–2^ were performed using MP Fastprep 24 (Mp Biomedical). In between the cycles, samples were placed on ice for 5 min. Tubes were centrifuged at 12,000 *g* for 10 min at 4°C and the supernatants were collected and mixed with chloroform to obtain phase separation. The upper aqueous phase containing RNA was purified and concentrated with RNA Clean&Concentrator-5 (Zymo research). DNA elimination was performed by incubation of samples with 20 U of Turbo DNase (Ambion), followed by a second cycle of RNA Clean&Concentrator-5. RNA integrity was confirmed using the Agilent RNA 6000 Pico Kit (Agilent Technologies). Reverse transcription was performed using SuperScript III First-Strand Synthesis SuperMix (Invitrogen). After pre-amplification of the cDNA samples (12 pre-amplification cycles for bacterial cDNA and 14 cycles for mixed samples), quantitative real time PCR was performed using the high-throughput qRT-PCR Fluidigm Biomark HD. The transcription profile of 65 *S. aureus* virulence factors was assessed using specific TaqMan assays (see Supplementary Table 1; Brignoli et al., 2019). *gyrB* gene was used as the endogenous control to normalize the data. All the samples were normalized to inoculated bacteria using the ΔΔct method and presented as log_2_ (fold-change). The gene expression data obtained from three independent experiment showed good reproducibility (Pearson’s *r* > 0.6 for early time points and *r* > 0.9 for late time points; see Supplementary Figure 2). Due to the low levels of bacterial RNA obtained from the skin punches infected over 2 and 3 h (see Supplementary Figure 3), absolute quantification of two selected transcripts (*spa* and *hla*) was assessed by droplet digital PCR (BioRad), which showed to corroborate Fluidigm data (Supplementary Figure 4).

### Statistical Analysis

Statistical analysis was performed with GraphPad Prism v.8.0 software for one-way ANOVA and with SPSS v.25.0.0.2 to calculate Pearson correlation coefficients. Multiple array viewer (Mev) software was used for hierarchical clustering, using Pearson correlation as distance metric. Differences were considered significant at *P* < 0.05.

## Results

### Human Skin Explants Can Be Used to Study *Staphylococcus aureus* Skin Infection

To investigate colonization and infection of human skin by *S. aureus*, we first set up a model resembling the natural *S. aureus* cutaneous infections, using abdominal human skin explants. As shown in Figure 1, the uppermost layer of the epidermis (*stratum corneum*) was impaired by tape-stripping, to mimic skin abrasion, subsequently, the skin punches were inoculated with USA300 strain and cultured at air-liquid interface. We selected the community-acquired methicillin-resistant USA300 strain for this study because it is the predominant cause of SSTIs (King et al., 2006; Moran et al., 2006; Talan et al., 2011). To validate this model, we verified whether the non-infected skin punches could be cultured at air-liquid interface for up to 72 h without undergoing apoptosis, we compared the detection of active-caspase-3 (a marker for apoptosis) in fresh tissue, tissue cultured for over 72 h and for over 6 days, as a control. Fresh tissue and tissue cultured over 72 h did not express active-caspase-3, contrarily to tissue cultured up to 6 days (Supplementary Figure 1A), indicating that the skin punches can be incubated at air-liquid interface for at least 72 h. By comparing skin sections of non-stripped and stripped skin punches stained with anti-cytokeratin antibodies, we also demonstrated that the tape-stripping procedure effectively removed most of the *stratum corneum* (Supplementary Figure 1B). Finally, after infection of skin punches with GFP-expressing USA300 strain for 72 h, we performed two-photon microscopy and detected bacteria in the dermis layer, at different depths (Supplementary Figure 1C), confirming that *S. aureus* could invade the skin tissues from epidermis to the dermis. Overall, we optimized a human cutaneous infection model that is suitable to study *S. aureus* skin infections.

### *S. aureus* Virulence Factors Group Into Four Main Clusters According to Transcription Profile During Skin Infection

**FIGURE 2:**
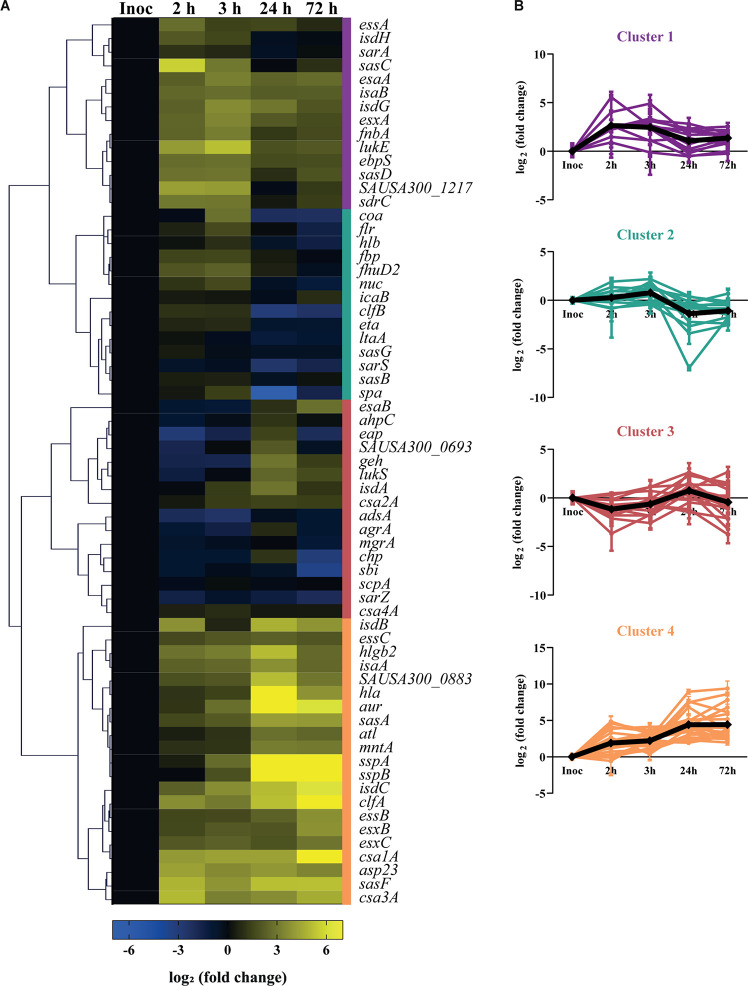
Virulence gene expression kinetics of USA300 during infection of human skin explants. **(A)** Heatmap showing the transcription profile of 65 virulence factors of *S. aureus* during cutaneous infection. Values represent the mean of three independent experiments, normalized to the transcription levels of the inoculated bacteria (exponential phase, OD_600_ = 0.6–0.7) and shown as log_2_ (fold change). The genes that were less or more transcribed in the skin model than in the inoculated bacteria are depicted in blue or yellow, respectively. The colored bars on the right side of the heatmap represent the four major clusters: cluster 1 (purple), cluster 2 (green), cluster 3 (red), cluster 4 (orange). **(B)** Transcript level variation over infection of the virulence factors grouped within the same cluster. The black line represents the mean profile of the cluster genes.

To monitor *S. aureus* virulence in human skin, we studied the gene expression of a large panel of virulence factors after 2 and 3 h of infection for colonization, and after 24 and 72 h for established infection. We selected 65 virulence-related genes that encode adhesion, invasion and immune evasion proteins (see Supplementary Table 1), that are normally expressed by the bacteria at different stages of infection. The heatmap shows the overall transcription kinetics of the selected genes during skin colonization and infection (Figure 2A).

Cluster analysis subdivided the analyzed genes into four main clusters, based on their transcription profile over the time-course infection (Figures 2A,B). Cluster 1 includes genes whose transcription increases upon skin contact and slightly decreases while infection progresses, like *sasC* and *sasD*, which encode for the *S. aureus* surface proteins C and D, respectively (Figures 2A,B; cluster 1). Cluster 2 includes the genes whose transcripts levels are similar with those of the inoculated bacteria during colonization but that decrease in expression after 24 h of skin infection. This cluster also includes genes that encode for surface proteins such as clumping factor B (*clfB*), staphylococcal protein A (*spa*) and *S. aureus* surface proteins G and B (*sasG, sasB*) (Figures 2A,B; cluster 2). Cluster 3 represents the genes that are downregulated early in infection but increase in expression after 24 h of infection, and includes genes that encode proteins that play a role in transcription regulation like *agrA*, *mgrA*, and *sarZ* (Figures 2A,B; cluster 3). Finally, cluster 4 contains the genes that are highly expressed over the entire course of infection, and includes many genes that encode secreted factors such as alpha-hemolysin (*hla*), aureolysin (*aur*) and the ESAT-6 secretion system (also known as Type VII secretion system, or T7SS) extracellular protein C (*esxC*) (Figures 2A,B; cluster 4). Taken together, these data show the transcription kinetics of 65 virulence genes of USA300 during colonization and infection of human skin.

### Bacteria Modulate Virulence Gene Expression During Skin Colonization and Infection

**FIGURE 3:**
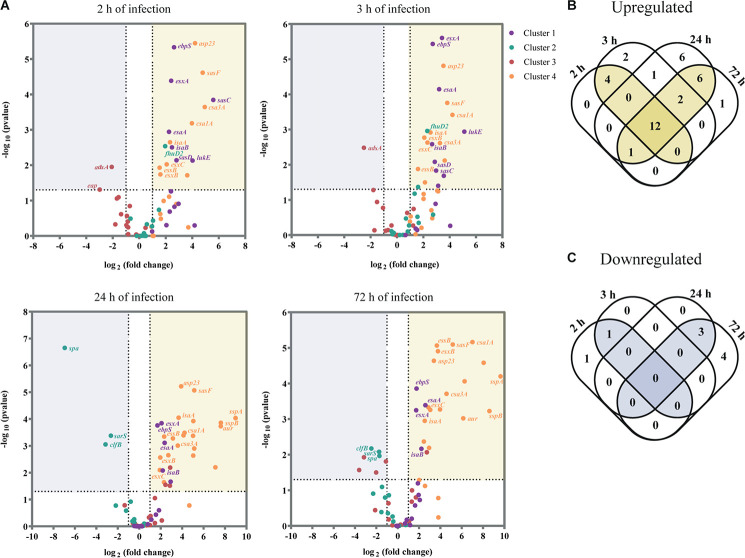
USA300 virulence factors differentially expressed during *ex vivo* skin infection when compared with inoculated bacteria. **(A)** Volcano plot showing the transcripts that are significantly downregulated (≥2-fold decrease, *P* < 0.05) or upregulated (≥2-fold increase, *P* < 0.05) for each time point (2, 3, 24, and 72 h of infection). Data were normalized over the inoculated bacteria and are expressed in log_2_ (fold change). The results are shown as the mean of three independent experiments. For each assay, statistical analysis was performed by one-way ANOVA, with Tukey’s multiple comparison tests. **(B,C)** Venn diagram showing the number of virulence factors that are upregulated **(B)** and downregulated **(C)** per time point.

Having shown the transcription kinetics of the selected virulence factors within human skin tissues, we next identified the factors that were significantly up or downregulated during infection by at least twofold, relative to their expression level in inoculated bacteria (*P* < 0.05; Figure 3A, see Supplementary Table 2). As a strong correlation was observed between the transcription profile of *S. aureus* virulence factors at early time points (after 2 and 3 h of skin infection) and also the two late time points (24 and 72 h of infection) (Pearson’s *r* > 0.8; see Supplementary Figure 5), we considered the early time points as early skin infection/colonization and the late time points as late infection.

Of the 65 virulence factors, 16 genes were significantly upregulated at early infection/colonization while 1 gene was downregulated (Figures 3B,C; see Supplementary Table 3). As anticipated, most of these genes encode for virulence factors that are associated with tissue colonization and evasion of host defenses. Namely, genes that encode surface proteins as the elastin binding protein (*ebpS*) and the *S. aureus* surface proteins C, D and F (*sasC*, *sasD*, and *sasF*) were upregulated upon skin contact. However, we also identified as upregulated at colonization stage the ferric hydroxamate uptake D2 lipoprotein (*fhuD2*) that is involved in iron uptake and in early stages of invasive *S. aureus* infection (Mishra et al., 2012) and Leukotoxin ED (*lukE*), responsible for neutrophil killing (Alonzo et al., 2012). The immune evasion protein adenosine synthase A (*adsA*), was the only gene identified as significantly downregulated at both 2 and 3 h of infection.

Later in infection, 21 genes were significantly upregulated and 3 were downregulated (Figures 3B,C; see Supplementary Table 3). We verified that most of the upregulated genes are secreted factors as toxins and proteases while the downregulated genes are associated to adhesion to host tissue and immune evasion. For example, V8 protease (*sspA*), staphopain B (*sspB*), and aureolysin (*aur*), three major secreted proteolytic enzymes of *S. aureus* were all significantly upregulated at this stage. On the other hand, the genes that encode the immune evasion protein SpA and the adhesion protein ClfB were significantly downregulated at late infection, as well as the transcriptional regulator *sarS*, which represses the expression of *spa* (Cheung et al., 2001).

Remarkably, 12 virulence factors were significantly upregulated over the entire course of the infection (Figure 3B and Table 1). These 12 genes encode for four proteins that are part of the ESAT-6 secretion system (*esxA*, *esxB*, *esxC*, *esaA*, and *essB*), two immunodominant antigens (*isaA* and *isaB*), two proteins from the family of conserved staphylococcal antigens (*csa1A* and *csa2*), two adhesion proteins (*ebpS* and *sasF)* and the alkaline shock protein 23 (*asp23*).

**TABLE 1:** Virulence gene expression profile of the 12 factors that were upregulated over the entire course of the infection, when compared with inoculated bacteria.

**Gene name**	**log_2_ (fold change)**			
	**2 h**	**3 h**	**24 h**	**72 h**
Alkaline shock protein 23 (*asp23*)	4.21	3.53	3.91	3.39
ESAT-6 secretion acessory factor A (*esaA*)	2.24	3.20	2.37	2.59
ESAT-6 secretion machinery protein B (*essB*)	1.53	1.59	2.32	3.67
ESAT-6 secretion system extracellular protein A (*esxA*)	2.41	3.42	2.08	1.73
ESAT-6 secretion system extracellular protein B (*esxB*)	1.59	2.08	1.96	3.77
ESAT-6 secretion system extracellular protein C (*esxC*)	2.07	2.31	1.92	2.89
Elastin-binding protein (*ebpS*)	2.62	2.71	1.70	1.77
Immunodominant antigen A (*isaA*)	2.32	2.54	3.67	2.53
Immunodominant antigen B (*isaB*)	2.47	2.67	2.19	2.22
Conserved staphylococcal antigen 1A (*csa1A*)	3.97	4.21	4.22	7.00
Conserved staphylococcal antigen 3A (*csa3A*)	4.94	3.26	3.59	4.57
*S. aureus* surface protein F (*sasF*)	4.79	3.82	5.13	5.16

Of note, when comparing the relative gene expression profile of *S. aureus* within skin tissue with that of *S. aureus* grown in broth medium, a weak correlation was observed for most of the time points assessed (see Supplementary Figure 6). Taken together, we observed that several genes were differently regulated within the skin tissues when compared with the initial inoculum, which likely reflects their importance for colonization and infection of human skin.

### *S. aureus* Accumulates in Sweat Glands and *hla* Promoter Is Activated Within This Niche

**FIGURE 4:**
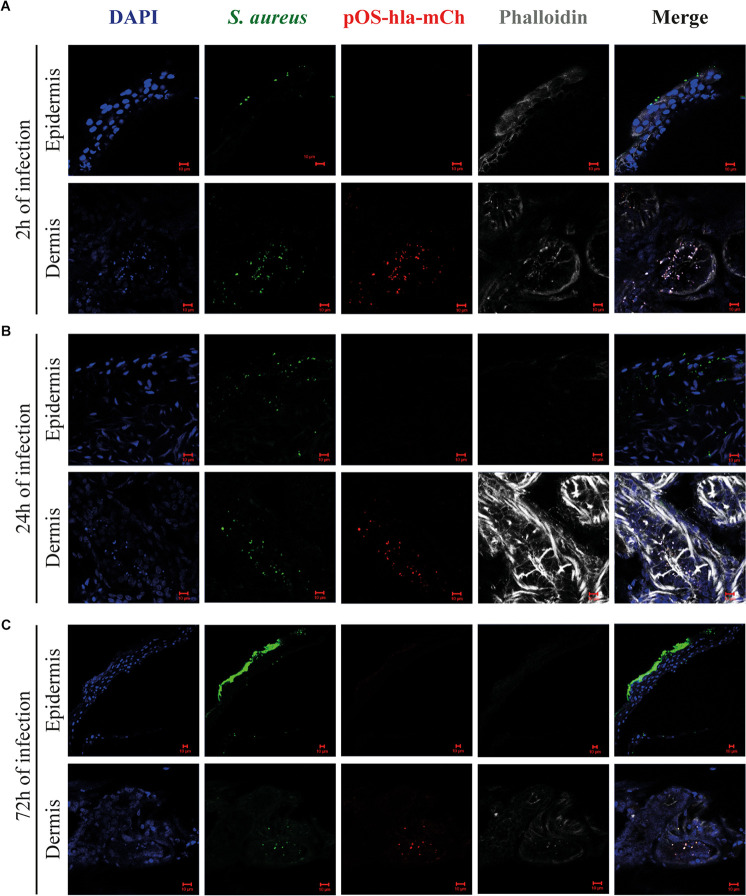
Alpha hemolysin reporter system activation within human skin tissues. Representative images of cryosections of skin infected with *S. aureus* (pOS-hla-mCherry, red), stained with anti-*Staphylococcus aureus* antibody (green), phalloidin (gray) and DAPI (blue) after 2 **(A)**, 24 **(B)** and 72 h of infection **(C)**. mages were taken at the epidermis (upper images) and the dermis (lower images) (scale bar, 10 μm).

Having studied the overall virulence gene expression of bacteria within a human skin punch, we next verified whether *S. aureus* location in the skin tissues could influence virulence gene transcription. Following skin abrasion, skin punches were infected with a USA300 reporter fusion strain, where the promoter of alpha hemolysin gene was fused together with the *mCherry* gene, which enables direct visualization of differences in *hla* expression *in situ*. The infection was monitored by confocal microscopy after 2, 24, and 72 h (Figure 4). After only 2 h, *S. aureus* preferentially accumulated on the skin surface and inside sweat glands and ducts (Figure 4A). Remarkably, after 2 h of infection, *hla* promoter was activated inside the sweat glands and ducts but not on the skin surface, where the infection begun (Figure 4A). mCherry signal was also detected inside these skin appendices after 24 and 72 h of infection, but not at the skin surface (Figures 4B,C). Altogether, these data show that *S. aureus* accumulates inside sweat glands and ducts and suggests that *S. aureus* niches within the skin tissues influences its transcription profile.

## Discussion

*Staphylococcus aureus* is the predominant cause of SSTIs (McCaig et al., 2006). Its success as a pathogen is largely dependent on its ability to adapt to different environments by modulating the expression of cell surface-associated and secreted factors that promote adherence, immune evasion and invasion of host tissues. To better understand staphylococcal pathogenesis in human tissues, it is important to determine which bacterial virulence factors are expressed in the context of the infection. Therefore, in this study we established a *S. aureus* cutaneous infection model using human skin explants and examined the gene expression profile of a vast array of *S. aureus* virulence factors at different stages of cutaneous infection.

We identified four main clusters of genes that respond similarly to environmental and host-specific signals during infection. In general, cluster 1 and 2 include the virulence factors that are more expressed by the bacteria early in infection. These clusters mostly comprise surface proteins that might be essential for human skin colonization. On the other hand, cluster 3 and 4 comprise the genes whose transcripts are increased later in infection and hence are likely important for skin invasion and long term persistence of infection. These clusters include genes that are involved in gene expression regulation and many genes that encode immune evasion proteins, toxins and secreted proteases. The fact that most adhesins are expressed in the beginning of infection and virulence-related genes are more expressed later in infection suggests that agr quorum sensing plays an important role in transcription regulation in human tissues, which is in accordance with a previous publication where the gene expression of a small panel of *S. aureus* virulence factors was analyzed in samples collected from human cutaneous abscesses (Loughman et al., 2009). Nevertheless, we detected only a weak correlation between the relative gene expression pattern of USA300 grown *in vitro* vs. in skin explants for most time points analyzed, which underlines the importance of environmental and host-specific cues for *S. aureus* gene expression regulation. An example is the upregulation of *fhuD2* early in skin infection. This upregulation is likely a consequence of the iron-limiting conditions that *S. aureus* faces upon contact with human tissues, as the concentration of free iron *in vivo* is very limited when compared to laboratory media (Ratledge and Dover, 2000; Hazmanian et al., 2003).

In this study, we also pinpointed 12 genes that were upregulated over the entire course of the infection when comparing with inoculated bacteria. *EsxA*, *esxB*, *esxC*, *esaA*, and *essB* are part the Ess pathway that contributes for *S. aureus* virulence (Burts et al., 2005) and establishment of persistent infection (Burts et al., 2008; Anderson et al., 2011). The overexpression of Ess genes upon skin contact may be explained by bacterial interaction with host-specific fatty acids, which were shown to activate Ess operon (Lopez et al., 2017). The transcript levels of the immunodominant antigens *isaA* and *isaB* were also enhanced during skin infection. High titers of IgG against both proteins were detected in sera from sepsis patients, indicating that *S. aureus* expresses IsaA and IsaB during human infection (Lorenz et al., 2000). The genes that encode SasF and EbpS were also upregulated over infection. SasF is described to protect the bacteria from bactericidal effects of long-chain unsaturated fatty acids (Kenny et al., 2009) and was also shown to be involved in *S. aureus* virulence in mice skin abscess infections (Kwiecinski et al., 2014), therefore, its upregulation might occur as a *S. aureus* defense mechanism. Although the role of EbpS has not been implicated in *S. aureus* skin infections, by binding to elastin, EbpS may promote colonization of skin tissues that are rich in elastin (Downer et al., 2002). The gene that encodes the A*sp23* was also highly expressed in the skin tissues. Asp23 is involved in cell homeostasis (Müller et al., 2014) and was identified as a CD4 T Cell antigen (Lawrence et al., 2012) but, as EbpS, it was not associated before to skin infection. Also *csa1A* and *csa3A* genes, which are members of the conserved staphylococcal antigens family that was recently identified to be involved in biofilm formation (Kavanaugh et al., 2019), were upregulated over infection. Although the effect of Csa1A and Csa3A were not studied alone in skin tissues, a four-component *S. aureus* vaccine (Hla, EsxA-EsxB, FhuD2, and Csa1A) showed to reduce abscess formation, CFU counts and dermonecrosis (Bagnoli et al., 2015).

Further studies are needed to clarify which stimulus result in a specific alteration in the gene expression of the bacteria and to pinpoint the factors that are essential for colonization and infection. The use of mutant strains lacking the expression of a global regulator to infect the *ex vivo* model presented here may aid the understanding of gene expression regulation in human tissues. In addition, it would be of interest to compare the gene expression pattern of USA300 LAC strain in human skin with other *S. aureus* strains. Importantly, although human skin explants contain all cells types found in skin tissues, including immune cells (Nestle et al., 2009), they lack the presence of neutrophils. Neutrophils play an important role during *S. aureus* skin infections, as these cells are recruited from blood to the site of infection for bacterial clearance (Miller and Cho, 2011; Kobayashi et al., 2015). Therefore, the explant model used here may not perfectly mimic the micro-environment encountered by the bacteria in human skin. Future developments in the field of microfluidic skin-on-a-chip devices may enable culture of the human skin tissues in presence of a blood flow and help to further improve the skin model used in this study.

We also observed that *S. aureus* accumulates inside sweat glands and ducts. The visualization of staphylococci biofilm occluding sweat glands was previously reported in atopic dermatitis skin samples (Allen et al., 2014) and also in miliaria (retention of eccrine sweat caused by blockage of sweat ducts) (Mowad et al., 1995). Additionally, although the relationship between hidradenitis suppurativa (chronic inflammatory skin disease) and bacterial infections is still controversial, *S. aureus* and coagulase-negative staphylococci have been reported among the most prevalent isolates detected (Ring et al., 2015; Ring and Emtestam, 2016). Therefore, skin appendices might represent a preferential niche for colonization/infection. Eccrine sweat glands are known for their function in thermoregulation but also for being part of innate immune defense of human skin against infection, due to production of the antimicrobial peptide dermcidin (DCD). DCD is specifically and constitutively expressed in eccrine sweat glands and secreted in the sweat (Schittek et al., 2001). Interestingly, DCD was shown to upregulate *Staphylococcus epidermidis* agr system and downregulate SarA and SaeRS regulators (Lai et al., 2007), inducing protease expression. Therefore, although the effect of DCD on *S. aureus* agr system was not assessed, exposure to DCD in a sweat glands and ducts might affect *hla* expression. Besides, the environment cues characteristic of the sweat gland, as its acidic pH (4–6.8) and high salt concentrations, can influence *S. aureus* virulence regulation.

Overall, our work shows for the first time the kinetics of *S. aureus* virulence gene expression within human skin and also suggests that sweat glands and ducts are a preferential niche for *S. aureus* colonization, where the virulence-related gene *hla* is more expressed. Increasing knowledge about gene expression profile within the target tissue is key for the design of new therapeutics to control infections caused by *S. aureus*.

## Data Availability Statement

The raw data supporting the conclusions of this article will be made available by the authors, without undue reservation.

## Ethics Statement

The studies involving human participants were reviewed and approved by the French Ministry of Higher Education, Research and Innovation. The patients/participants provided their written informed consent to participate in this study.

## Author Contributions

AC, JS, FB, and AM were involved in designing or conceiving the study. AC performed experiments and analyzed the data. AC and AM wrote the manuscript. JS and FB acquired the funding. FB and AM supervised the project. All authors critically revised the manuscript and approved it before submission.

## Conflict of Interest

AC participated in a postgraduate studentship program at GSK. FB and AM are employees of GSK group of companies and FB holds shares in the GSK group of companies. FB and AM were named inventors of pending and issued patents on *S. aureus* vaccine formulations. The remaining author declares that the research was conducted in the absence of any commercial or financial relationships that could be construed as a potential conflict of interest.
