# Supplementary material for: Virulence Gene Expression of Staphylococcus aureus in Human Skin
Source: Front Microbiol. 2021 Jun 11;12:692023. doi: 10.3389/fmicb.2021.692023 (PMC8231915; doi:10.3389/fmicb.2021.692023)
Supplement: Supplementary file 1 [file Data_Sheet_1.docx]

Supplementary Material

# Supplementary Figures and Tables

**
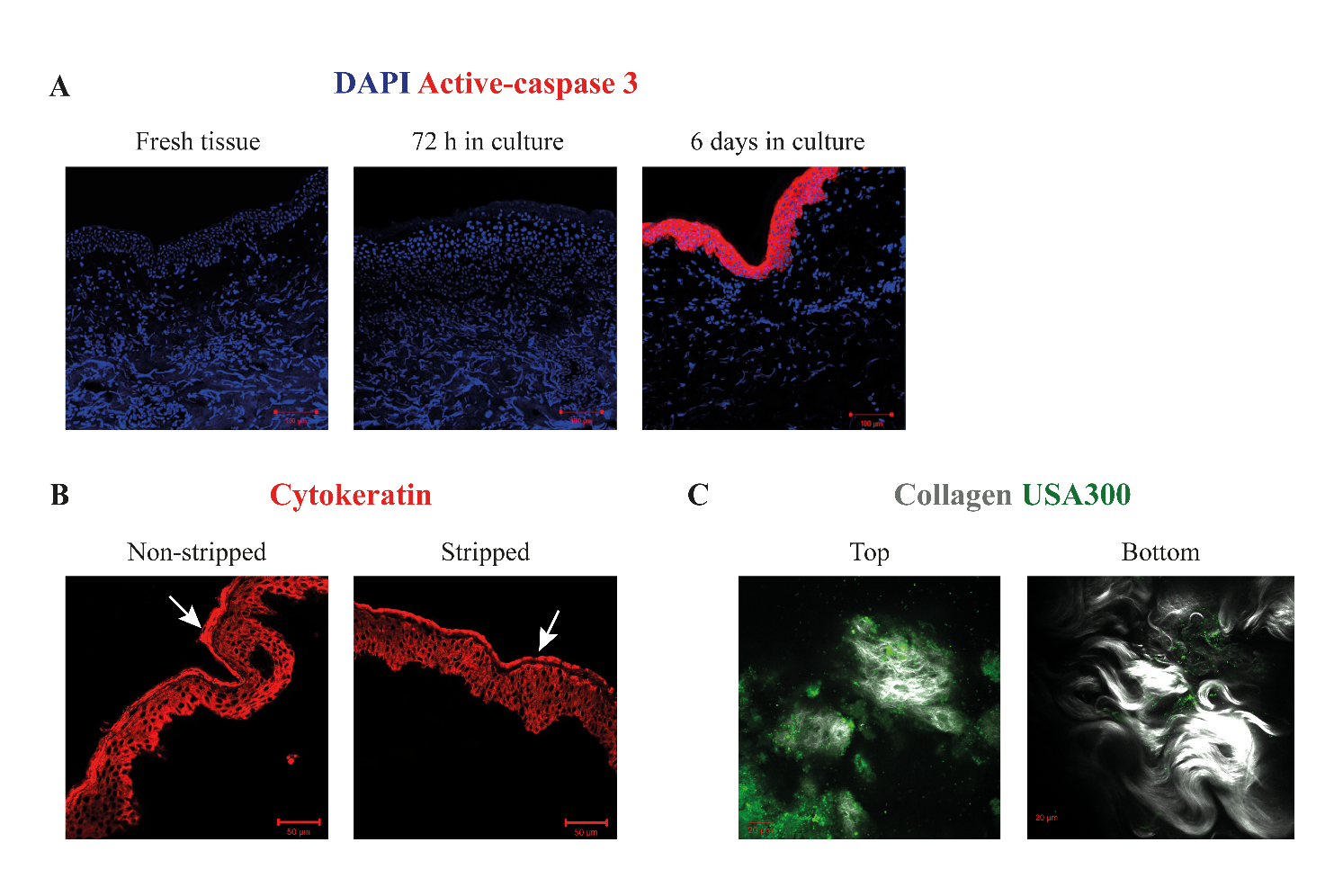
**

**Supplementary Figure 1.** **Validation of the *S. aureus* cutaneous infection model.** **(A)** Confocal microscopy images of skin cryosections stained with anti-active-caspase 3 antibody and DAPI at time 0 (fresh tissue), after 72 hours and 6 days in culture (scale bar, 100 µm). **(B)** Confocal microscopy images of cryosections of intact skin punches (non-stripped) and of punches submitted to the tape-stripping method (stripped), stained with anti-wide spectrum cytokeratin antibody (scale bar, 50 µm). The white arrows indicate the uppermost layer of the epidermis (*stratum corneum*). **(C)** Two-photon microscopy images of a skin punch infected with GFP-expressing USA300 LAC strain for 72 hours, taken at a depth of approximately 200 µm from epidermis to the dermis (left image) and of approximately 400 µm from the dermis to the epidermis (right image) (scale bar, 20 µm).

**
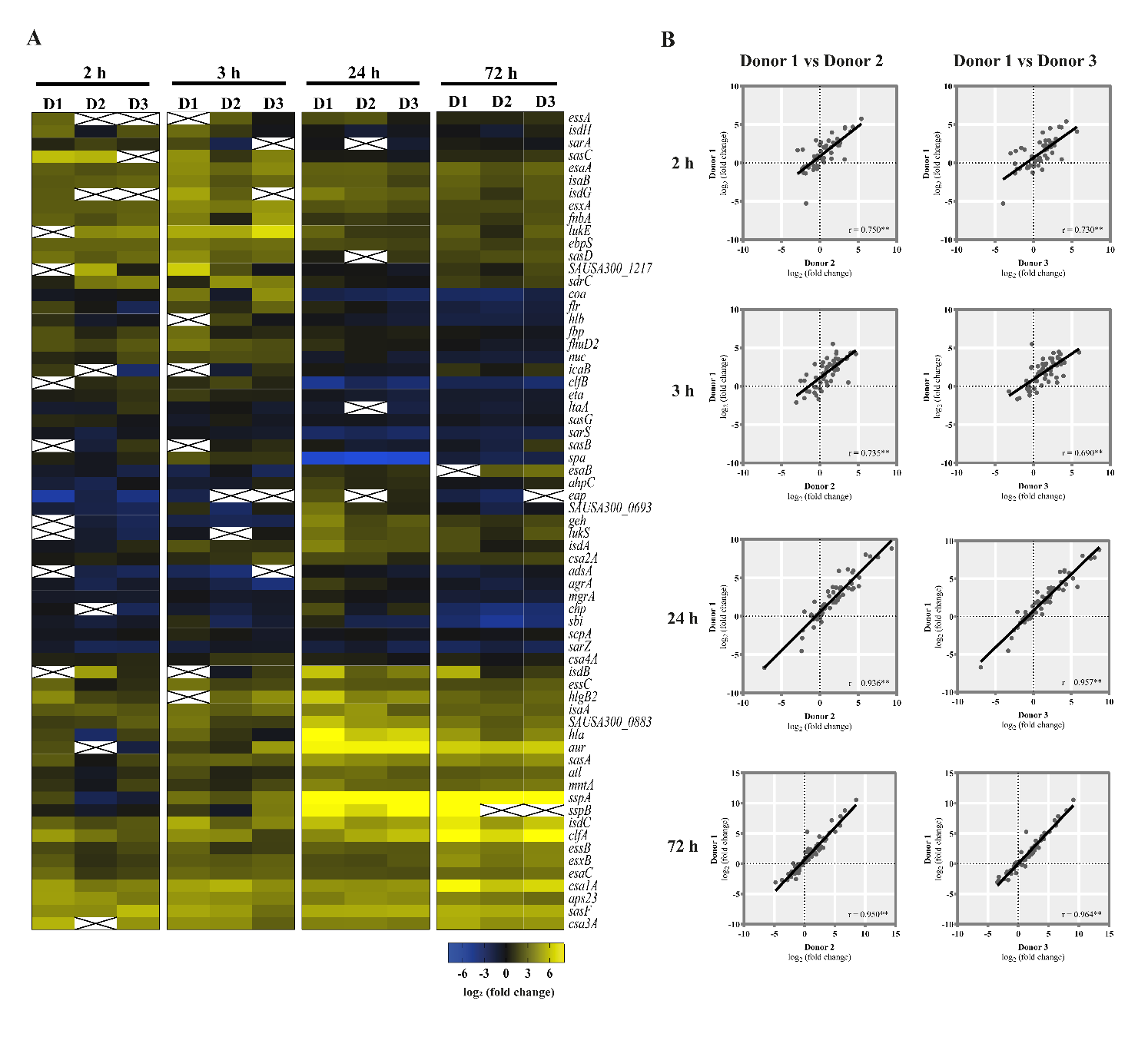
**

**Supplementary Figure 2. Virulence gene expression kinetics of USA300 during infection of human skin explants collected from three different donors. (A)** Heatmap showing the transcription profile of 65 virulence factors of *S. aureus* during cutaneous infection of three donors (D1, D2, D3). Data were normalized over the transcription levels of the inoculated bacteria (exponential phase, OD_600_ 0.6-0.7) and shown as log_2_ (fold change). The genes that were less or more transcribed in the skin model than in the inoculated bacteria are depicted in blue or yellow, respectively. **(B)** Pairwise correlation between the gene expression profile of *S. aureus* during infection of skin explants collected from three different donors. Pearson correlation coefficients are shown in the right bottom corner of graphs (** *P* < 0.01).

**
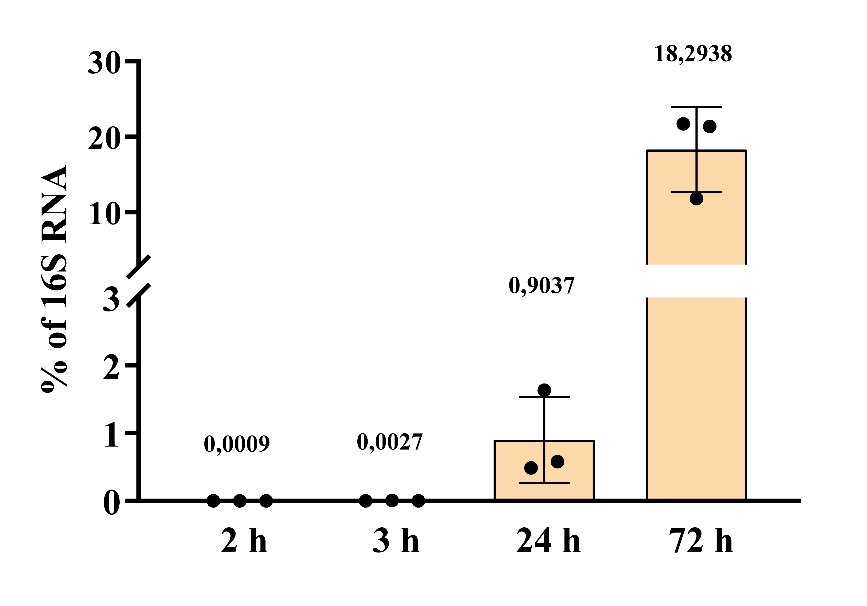
**

**Supplementary Figure 3. Percentage of bacterial RNA in human skin samples infected with *S. aureus*.** Percentage of 16S ribosomal RNA in skin tissues infected over 2, 3, 24 and 72 hours with *S. aureus* USA300 LAC strain. Data represent the mean ± SD of three independent experiments.


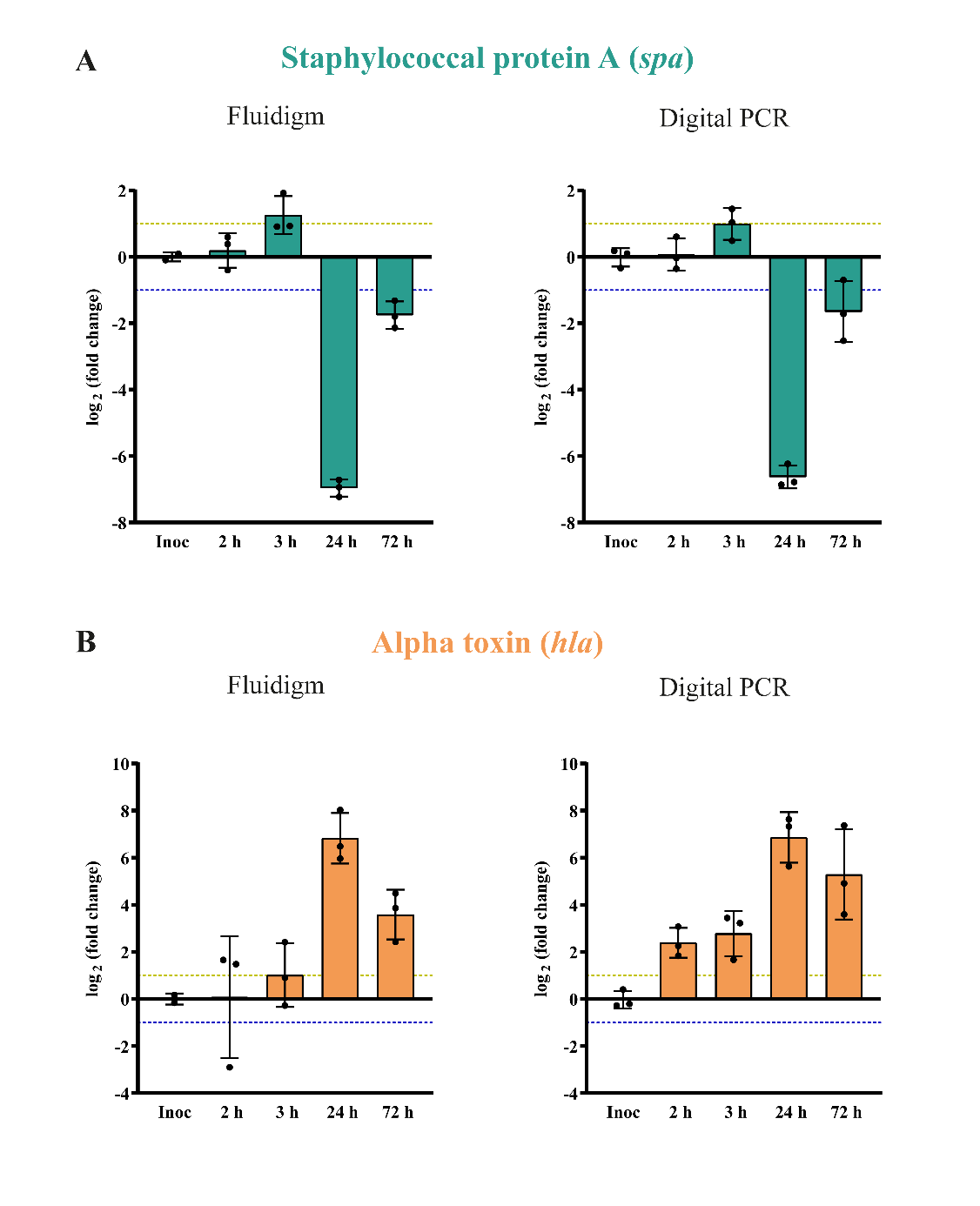


**Supplementary Figure 4. Validation of transcript levels obtained with Fluidigm by digital PCR.** **(A, B)** Gene expression profile of *spa* **(A)** and *hla* **(B)** within the skin tissue over time, detected by Fluidigm (left graphs) and digital PCR (right graphs). Data were normalized over the inoculated bacteria and presented in log_2_ (fold-change). The dotted lines represent a 2-fold increase or decrease. The results are shown as the mean ± SD of two or three independent experiments.


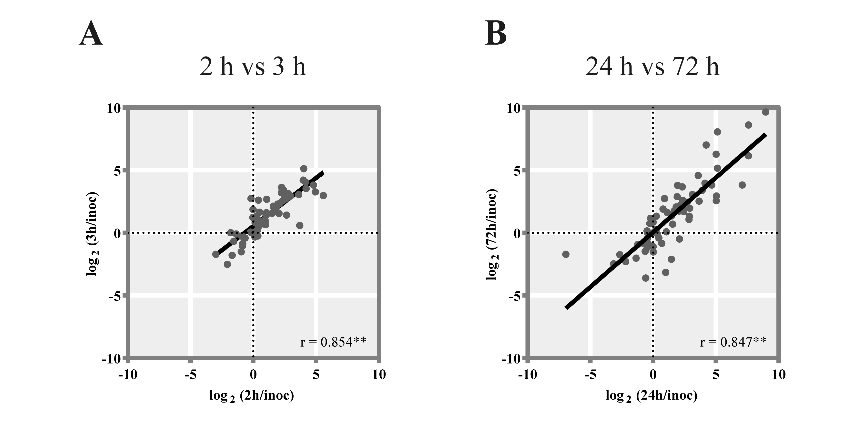


**Supplementary Figure 5. Pairwise correlation between virulence gene expression of USA300 within human skin explants at early and late infection stages.** Pearson correlation coefficients are shown in the right bottom corner of graphs (***P <* 0.01).


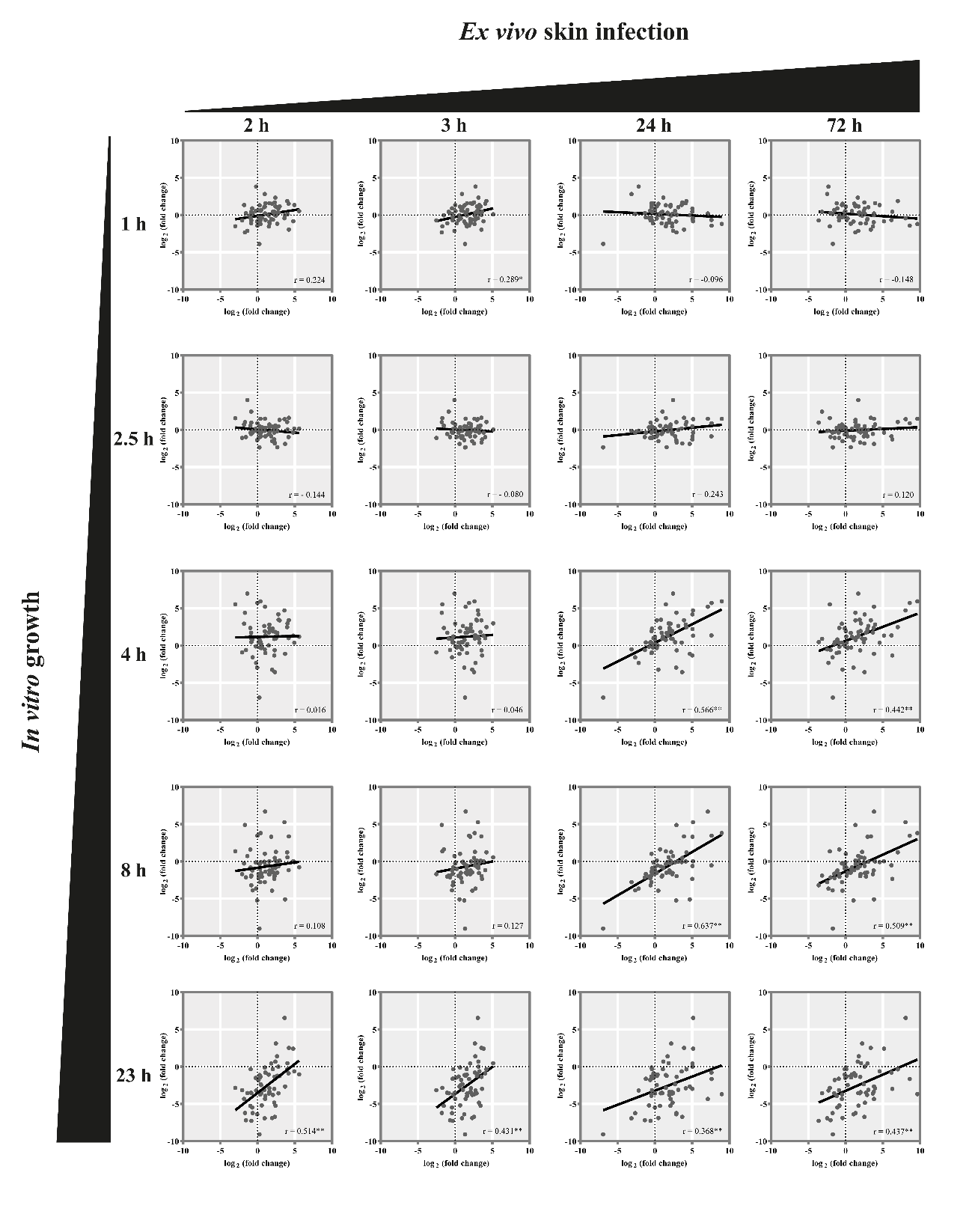


**Supplementary Figure 6. Pairwise correlation between virulence gene expression of USA300 during infection of human skin explants and during in vitro growth in broth medium**. Pearson correlation coefficients are shown in the right bottom corner of graphs (**P <* 0.05; ***P* < 0.01).

**Supplementary Table 1.** TaqMan assays used in this study [1].

| **Assay name** | | | **USA300 gene** | | | | **Gene name** | | | **Oligo function** | | | | **Sequence** | | | | |  |  |  |
| --- | --- | --- | --- | --- | --- | --- | --- | --- | --- | --- | --- | --- | --- | --- | --- | --- | --- | --- | --- | --- | --- |
| *adsA* | | | SAUSA300_0025 | | | | Adenosine synthase A | | | Forward primer  Reverse primer  Probe | | | | CATCACTTTCACCACGAATGTTTG  GCGTCTAACATTAAATCAGGCTTTTC | | | | |  |  |  |
|  |  |  |  |  |  |  |  |  |  |  |  |  |  | TTACATACAAATGATATCC | | | | |  |  |  |
| *agrA* | | | SAUSA300_1992 | | | | Accessory gene regulator protein A | | | Forward primer | | | | CTCGCAACTGATAATCCTTATGAGG | | | | |  |  |  |
|  |  |  |  |  |  |  |  |  |  | Reverse primer | | | | GTAACGAAAATAATGTTACCAACTGGG | | | | |  |  |  |
|  |  |  |  |  |  |  |  |  |  | Probe | | | | GATATTCAACTTTCAACTG | | | | |  |  |  |
| *ahpC* | | | SAUSA300_0380 | | | | Alkyl hydroperoxide reductase subunit C | | | Forward primer | | | | GAAATCTTACCATTTACAGCGCAAG | | | | |  |  |  |
|  |  |  |  |  |  |  |  |  |  | Reverse primer | | | | GCCTAATTTTTGTAATTCTTCATATTGG | | | | |  |  |  |
|  |  |  |  |  |  |  |  |  |  | Probe | | | | CTATCCTGCTGACTTCTC | | | | |  |  |  |
| *asp23* | | | SAUSA300_2142 | | | | Alkaline shock protein 23 | | | Forward primer | | | | CAAGCATACGACAATCAAACTGGTG | | | | |  |  |  |
|  |  |  |  |  |  |  |  |  |  | Reverse primer | | | | GCAGCGATACCAGCAATTTTTTC | | | | |  |  |  |
|  |  |  |  |  |  |  |  |  |  | Probe | | | | CGTCAAAAACAACAAGAAC | | | | |  |  |  |
| *atl* | | | SAUSA300_0955 | | | | Autolysin | | | Forward primer | | | | CGAAACAGCACCAACGGATTACTTA | | | | |  |  |  |
|  |  |  |  |  |  |  |  |  |  | Reverse primer | | | | CAGCATAGTTATTCATTGAACGTGCAA | | | | |  |  |  |
|  |  |  |  |  |  |  |  |  |  | Probe | | | | ACTGCACCGACACCC | | | | |  |  |  |
| *aur* | | | SAUSA300_2572 | | | | Aureolysin | | | Forward primer | | | | GAGCACTTTATCACCAGCAGCATTAG | | | | |  |  |  |
|  |  |  |  |  |  |  |  |  |  | Reverse primer | | | | GTTTTTACATCAGTAACAGCGTAATCTTG | | | | |  |  |  |
|  |  |  |  |  |  |  |  |  |  | Probe | | | | GAGGTGACTCAAAAGAG | | | | |  |  |  |
| *chp* | | | SAUSA300_1920 | | | | Chemotaxis-inhibiting protein CHIPS | | | Forward primer | | | | GGAATCAGTACACACCATCATTCAG | | | | |  |  |  |
|  |  |  |  |  |  |  |  |  |  | Reverse primer | | | | ATTTCTCAAACGTTCATCTAATTTTCC | | | | |  |  |  |
|  |  |  |  |  |  |  |  |  |  | Probe | | | | CCGTTTCCTACAAATG | | | | |  |  |  |
| *clfA* | | | SAUSA300_0772 | | | |  | | | Forward primer | | | | CAACGAATCAAGCTAATACACCG | | | | |  |  |  |
|  |  |  |  |  |  |  | Clumping factor A | | | Reverse primer | | | | GTTGTTGAAACATTTTCCGCATTTG | | | | |  |  |  |
|  |  |  |  |  |  |  |  | | | Probe | | | | GTGAATCAAACAAGTAATG | | | | |  |  |  |
| *clfB* | | | SAUSA300_2565 | | | | Clumping factor B | | | Forward primer | | | | GGATAGGCAATCATCAAGCACAAG | | | | |  |  |  |
|  |  |  |  |  |  |  |  |  |  | Reverse primer | | | | GCTATCTACATTCGCACTGTTTGTG | | | | |  |  |  |
|  |  |  |  |  |  |  |  |  |  | Probe | | | | CAATATGATAGAAACACC | | | | |  |  |  |
| *coa* | | | SAUSA300_0224 | | | | Coagulase | | | Forward primer | | | | GAAATAAAACCACAAGGTACTGAATCAACG | | | | |  |  |  |
|  |  |  |  |  |  |  |  |  |  | Reverse primer | | | | GCTTCATATCCAAATGTTCCATCG | | | | |  |  |  |
|  |  |  |  |  |  |  |  |  |  | Probe | | | | CAATTTAACAAAACACC | | | | |  |  |  |
| csa1A | | | SAUSA300_0100 | | | | Conserved staphylococcal antigen 1A | | | Forward primer  Reverse primer  Probe | | | | GAAGATATGGTAGCTAAAGGCATGGTTC  GTGCGGTTTTCCTTCATCC | | | | |  |  |  |
|  |  |  |  |  |  |  |  |  |  |  |  |  |  | CTACTATGTCGATGTGACT | | | | |  |  |  |
| *csa2A* | | | SAUSA300_0205 | | | | Conserved staphylococcal antigen 2A | | | Forward primer  Reverse primer  Probe | | | | CGCTTTATGAAATTGATGGTCACG  CTTATCTACCTTTAACAACATACGGTCTTC | | | | |  |  |  |
|  |  |  |  |  |  |  |  |  |  |  |  |  |  | CAGTTGTTGGATCAGATG | | | | |  |  |  |
| *csa3A* | | | | SAUSA300_0410 | | | | | Conserved staphylococcal antigen 3A | | | | Forward primer | | | GAACCGCTAAAGGCCATTATTTTGTTAC | |  |  |  |  |
|  |  |  |  |  |  |  |  |  |  |  |  |  | Reverse primer | | | CTGCTTGAGCTTATCATCTTTTACTTC | |  |  |  |  |
|  |  |  |  |  |  |  |  |  |  |  |  |  | Probe | | | CCGGAATGGTAAACTAC | |  |  |  |  |
| *csa4A* | | | | SAUSA300_2424 | | | | | Conserved staphylococcal antigen 4A | | | | Forward primer  Reverse primer  Probe | | | GAACCACAAAAGGGTATTATTTTATAAGTG  CTTGTCATTCGGTAGTGGCTTCG  GTAACGGCAGACCAAAG | |  |  |  |  |
| *eap* | | | | SAUSA300_1917 | | | | | MHC class II analog protein | | | | Forward primer  Reverse primer  Probe | | | CGAAAATAAAGCTAAAAGAAACTATCAAG  GCTTTCTTAGCATATTTTAAATCTTGTTCAC  CATTTTCAAATAAACCTTG | |  |  |  |  |
| *ebpS* | | | | SAUSA300_1370 | | | | | Elastin binding protein | | | | Forward primer  Reverse primer  Probe | | | AAAGGTGCAGCGATCGGT  GCGGCAGAAGCACTTTTACTTG  CTCCAGCCAAACCTG | |  |  |  |  |
| *esaA* | | | | SAUSA300_0279 | | | | | ESAT-6 secretion acessory factor A | | | | Forward primer | | | GTTGCTGAGTCTGGTTTGAAAAATGG | |  |  |  |  |
|  |  |  |  |  |  |  |  |  |  |  |  |  | Reverse primer | | | GATATTTTCGATGGTGTTTTAGCGTC | |  |  |  |  |
|  |  |  |  |  |  |  |  |  |  |  |  |  | Probe | | | CATGATTGTTATCCCAG | |  |  |  |  |
| *esaB* | | | | SAUSA300_0281 | | | | | ESAT-6 secretion accessory factor B | | | | Forward primer | | | CAGCACGTAAAAGTAACATTTGATTTTAC | |  |  |  |  |
|  |  |  |  |  |  |  |  |  |  |  |  |  | Reverse primer | | | TTTCAACAAGTAATTGACCTTTCGTC | |  |  |  |  |
|  |  |  |  |  |  |  |  |  |  |  |  |  | Probe | | | ATAATTACGGCACATATG | |  |  |  |  |
| *essA* | | | | SAUSA300_0280 | | | | | ESAT-6 secretion machinery protein A | | | | Forward primer | | | CGACTCGCTTGAATGAAACTAAAAAAGTG | |  |  |  |  |
|  |  |  |  |  |  |  |  |  |  |  |  |  | Reverse primer | | | CCCCTACAGACATCAAAATGTACG | |  |  |  |  |
|  |  |  |  |  |  |  |  |  |  |  |  |  | Probe | | | AAGACTTCGGAGAGTG | |  |  |  |  |
| *essB* | | | | SAUSA300_0282 | | | | | ESAT-6 secretion machinery protein B | | | | Forward primer | | | GATTCCTAAGTCTTCAATTAAACCAGAAC | |  |  |  |  |
|  |  |  |  |  |  |  |  |  |  |  |  |  | Reverse primer | | | GGTGTATGATTGTCATTAATGTCATAATG | |  |  |  |  |
|  |  |  |  |  |  |  |  |  |  |  |  |  | Probe | | | CCATATTTTATAGATGCTG | |  |  |  |  |
| *essC* | | | | SAUSA300_0283 | | | | | ESAT-6 secretion machinery protein C | | | | Forward primer | | | TTCGCCAAGGATTATTCACCGTG | |  |  |  |  |
|  |  |  |  |  |  |  |  |  |  |  |  |  | Reverse primer | | | CTAATGGCGGTATAATGGAACG | |  |  |  |  |
|  |  |  |  |  |  |  |  |  |  |  |  |  | Probe | | | CAATACAGAAGAACAATAC | |  |  |  |  |
| *esxA* | | | | SAUSA300_0278 | | | | | ESAT-6 secretion system extracellular protein A | | | | Forward primer | | | GAGTCCAGAGGAAATCAGAGCAAAA | |  |  |  |  |
|  |  |  |  |  |  |  |  |  |  |  |  |  | Reverse primer | | | CCTTGTGCACGTGTTAAATCAGATAAAA | |  |  |  |  |
|  |  |  |  |  |  |  |  |  |  |  |  |  | Probe | | | CTTGCCCGTAAGATTG | |  |  |  |  |
| *esxB* | | | | SAUSA300_0285 | | | | | ESAT-6 secretion system extracellular protein B | | | | Forward primer | | | TCGCTGAGTATATCGAAGGTAGTGA | |  |  |  |  |
|  |  |  |  |  |  |  |  |  |  |  |  |  | Reverse primer | | | CGGTTGTACTAATTCTTCTTGAAACTTTGC | |  |  |  |  |
|  |  |  |  |  |  |  |  |  |  |  |  |  | Probe | | | TTGGCGAACTGTCCTTC | |  |  |  |  |
| *esxC* | | | | SAUSA300_0284 | | | | | ESAT-6 secretion system extracellular protein C | | | | Forward primer  Reverse primer  Probe | | | GCTGAACAATATAAGCGATTAGAATTTAATTTGAGTT  TTAACAGTTACACTGTCCTTATTAGCACTA  CCTTTGCTGTGCTTTC | |  |  |  |  |
| *eta* | | | | SAUSA300_1065 | | | | | Exfoliative toxin A | | | | Forward primer | | | GCTTTCTTGATTTGGATTCACCTTTTATG | |  |  |  |  |
|  |  |  |  |  |  |  |  |  |  |  |  |  | Reverse primer | | | GCCAGACATGAAAAATGTTGTGAACAC | |  |  |  |  |
|  |  |  |  |  |  |  |  |  |  |  |  |  | Probe | | | AATAATGTGAAAGAACAAT | |  |  |  |  |
| *fbp* | | | SAUSA300_1101 | | | | | | Putative fibronectin/  fibrinogen binding protein | | | | Forward primer | | | | CACAAAATCAATCAACCTGATAATGAC |  |  |  |  |
|  |  |  |  |  |  |  |  |  |  |  |  |  | Reverse primer | | | | AATCTTGAAAAGTTTGGATGGATTGAC |  |  |  |  |
|  |  |  |  |  |  |  |  |  |  |  |  |  | Probe | | | | AAAATAGACAAAACCATC |  |  |  |  |
| *fhuD2* | | | SAUSA300_2235 | | | | | | Ferric hydroxamate receptor 2 | | | | Forward primer  Reverse primer  Probe | | | | CCAACAGTAGTTGTTGACTATAATAAGCA  GCAGTTGTTTCTTCCCAATCTTTCTT  CTTTACCAACAATTTTCC |  |  |  |  |
| *flr* | | | SAUSA300_1053 | | | | | | Formyl peptide receptor-like 1 inhibitory protein | | | | Forward primer  Reverse primer  Probe | | | | GCCAAGGTGATGTGAAGAAAGCAG  CGAGTCGATTTCACCGTTTTTAACAAC |  |  |  |  |
|  |  |  |  |  |  |  |  |  |  |  |  |  |  |  |  |  | GTCCTTTAGAAGAGAACAG |  |  |  |  |
| *fnbA* | | | SAUSA300_2441 | | | | | | Fibronectin binding protein A | | | | Forward primer | | | | ATTGAAACAATAGAAGAAACGGATTCATC |  |  |  |  |
|  |  |  |  |  |  |  |  |  |  |  |  |  | Reverse primer | | | | CTTCAAAGTCAATTGGATTTGATTCCTC |  |  |  |  |
|  |  |  |  |  |  |  |  |  |  |  |  |  | Probe | | | | CCATACTGCTGTGGATAG |  |  |  |  |
| *geh* | | | | SAUSA300_0320 | | | | | Triacylglycerol lipase | | | | Forward primer | | | CACATCAAATGCAGTCAGGAAAGC | |  |  |  |  |
|  |  |  |  |  |  |  |  |  |  |  |  |  | Reverse primer | | | CTTGTCGTTCAGAATCTTGCTTTACTTG | |  |  |  |  |
|  |  |  |  |  |  |  |  |  |  |  |  |  | Probe | | | GTGGAACAGTGACAGAAG | |  |  |  |  |
| *gyrB* | | | | SAUSA300_0005 | | | | | DNA gyrase subunit B | | | | Forward primer | | | GGTGACTGCATTGTCAGATGTAAAC | |  |  |  |  |
|  |  |  |  |  |  |  |  |  |  |  |  |  | Reverse primer | | | CTGCTTCTAAACCTTCTAATACTTGTATTTG | |  |  |  |  |
|  |  |  |  |  |  |  |  |  |  |  |  |  | Probe | | | CCCAGCACCATAATTA | |  |  |  |  |
| *hla* | | | | SAUSA300_1058 | | | | | Alpha-hemolysin | | | | Forward primer | | | TATAGTCAGCTCAGTAACAACAACA | |  |  |  |  |
|  |  |  |  |  |  |  |  |  |  |  |  |  | Reverse primer | | | TGCATGCCATTTTCTTTATCATAAGTGAC | |  |  |  |  |
|  |  |  |  |  |  |  |  |  |  |  |  |  | Probe | | | ATGCCGCAGATTCT | |  |  |  |  |
| *hlb* | | | | SAUSA300_1973 | | | | | Beta-hemolysin | | | | Forward primer | | | GTAATATTCAATGAAGCATTTGATAATGG | |  |  |  |  |
|  |  |  |  |  |  |  |  |  |  |  |  |  | Reverse primer | | | GGATATTTACTTACAATCGCTACGCC | |  |  |  |  |
|  |  |  |  |  |  |  |  |  |  |  |  |  | Probe | | | CTGAAGGTAGCTACTCATC | |  |  |  |  |
| *hlgB2* | | | | SAUSA300_2367 | | | | | Gamma-hemolysin, component B | | | | Forward primer | | | GCTACTGGGAATATTAACTCAGGCTTTG | |  |  |  |  |
|  |  |  |  |  |  |  |  |  |  |  |  |  | Reverse primer | | | GTGCATAATCAACGACGTTTACTGAATC | |  |  |  |  |
|  |  |  |  |  |  |  |  |  |  |  |  |  | Probe | | | GGGAGCTAAATACAATG | |  |  |  |  |
| *icaB* | | | | | | SAUSA300_2601 | | | Intercellular adhesion protein IcaB | | | | Forward primer | | | GGATGGTCATCATATTGCAAATGCA | |  |  |  |  |
|  |  |  |  |  |  |  |  |  |  |  |  |  | Reverse primer | | | AATTCGCTTTTCTTACACGGTGATAATTT | |  |  |  |  |
|  |  |  |  |  |  |  |  |  |  |  |  |  | Probe | | | CCAGAGCACTATTTTC | |  |  |  |  |
| *isaA* | | | | | | SAUSA300_2506 | | | Immunodominant antigen A | | | | Forward primer | | | CGTTGATCAAGCACACTTAGTTGACTTAG | |  |  |  |  |
|  |  |  |  |  |  |  |  |  |  |  |  |  | Reverse primer | | | GCTCCATGACCATGTAGTACCATTTGAAG | |  |  |  |  |
|  |  |  |  |  |  |  |  |  |  |  |  |  | Probe | | | CTCCAATCAAAGATGGTG | |  |  |  |  |
| *isaB* | | | | | | SAUSA300_2573 | | | Immunodominant antigen B | | | | Forward primer | | | GGCAAGGACTTGAAAAAAGAAAATGGT | |  |  |  |  |
|  |  |  |  |  |  |  |  |  |  |  |  |  | Reverse primer | | | CGACAACTCTATTATGATCAACGACAAAC | |  |  |  |  |
|  |  |  |  |  |  |  |  |  |  |  |  |  | Probe | | | ACCGCTATCAGCTTCC | |  |  |  |  |
| *isdA* | | | | | | SAUSA300_1029 | | | Iron transport associated domain-containing protein A | | | | Forward primer | | | GCAGTTGAACCTGGATATAAGAGCTTA | |  |  |  |  |
|  |  |  |  |  |  |  |  |  |  |  |  |  | Reverse primer | | | TGCTTTTTCAAATTCCAAATGCGTAGT | |  |  |  |  |
|  |  |  |  |  |  |  |  |  |  |  |  |  | Probe | | | TCGTGCCACAAATTAA | |  |  |  |  |
| *isdB* | | SAUSA300_1028 | | | | | | Iron transport associated domain-containing protein B | | | Forward primer  Reverse primer  Probe | | | | GGAGAAAATTTGAAGTTTATGAAGGTGACA  TGTTTTCGCTTTTTTATATGGCGCTAA  CAGTGCAGATAAATTC | | |  |  |  |  |
| *isdC* | | SAUSA300_1030 | | | | | | Iron transport associated domain-containing protein C | | | Forward primer  Reverse primer  Probe | | | | TAATTATCATCATCGCGACATTCAG  CCATTTTTCTTAATGTACTTTGCCGG  CAATACCAATGACACGTC | | |  |  |  |  |
| *isdG* | | SAUSA300_1035 | | | | | | Iron transport associated domain-containing protein G | | | Forward primer  Reverse primer  Probe | | | | CGAGACATGGGATTGAAACATTAGAAG  GGGCTACTTTCATCTTCATTTTTACTTC  ACAGTTTGGAAATCAAA | | |  |  |  |  |
| *isdH* | | SAUSA300_1677 | | | | | | Iron transport associated domain-containing protein H | | | Forward primer | | | | GTTGCATCGGTCATTGTCAGTAC | | |  |  |  |  |
|  |  |  |  |  |  |  |  |  |  |  | Reverse primer | | | | GTTGCATTATTATTTTGATTTTCCG | | |  |  |  |  |
|  |  |  |  |  |  |  |  |  |  |  | Probe | | | | CAAGCAGCAGAAAATAC | | |  |  |  |  |
| *ltaA* | | SAUSA300_0917 | | | | | | Probable glycolipid permease | | | Forward primer | | | | CTGTAGCAATAACGTCTCTAGCATTTTC | | |  |  |  |  |
|  |  |  |  |  |  |  |  |  |  |  | Reverse primer | | | | GGTGATGCTGGAAACCAAATAAC | | |  |  |  |  |
|  |  |  |  |  |  |  |  |  |  |  | Probe | | | | CAAAAATCGTTTTAACG | | |  |  |  |  |
| *lukE* | | SAUSA300_1769 | | | | | |  | | | Forward primer | | | | GATGTTGGTCAAACATTAGGATATAACATTG | | |  |  |  |  |
|  |  |  |  |  |  |  |  | Leukotoxin | | | Reverse primer | | | | ATTGTTTTAGAATAATTAAATGAGCCATTGCCA | | |  |  |  |  |
|  |  |  |  |  |  |  |  |  | | | Probe | | | | CTGACTGGAAATTACC | | |  |  |  |  |
| *lukS* | | SAUSA300_1975 | | | | | | Leukocidin | | | Forward primer | | | | GCAGCAACGACTCAAGCAAATTC | | |  |  |  |  |
|  |  |  |  |  |  |  |  |  |  |  | Reverse primer | | | | GTTTCAGTTCGTTTTGTGATTTTACCG | | |  |  |  |  |
|  |  |  |  |  |  |  |  |  |  |  | Probe | | | | GAACATGTTGATAAGTCTC | | |  |  |  |  |
| *mgrA* | | SAUSA300_0672 | | | | | | MarR family regulatory protein | | | Forward primer | | | | GCTCAAAGACAAGTTAATCGCTACTACTC | | |  |  |  |  |
|  |  |  |  |  |  |  |  |  |  |  | Reverse primer | | | | GTGCTAATTCAGTTACGACTTTCTTGAC | | |  |  |  |  |
|  |  |  |  |  |  |  |  |  |  |  | Probe | | | | CCCACAATTTCTTGTC | | |  |  |  |  |
| *mntA* | | SAUSA300_0620 | | | | | | ABC transporter ATP-binding protein | | | Forward primer | | | | GTGTGGAACAAGTGATTTTATCAGG | | |  |  |  |  |
|  |  |  |  |  |  |  |  |  |  |  | Reverse primer | | | | CACTTAATTCTGAAATTTGTCGATGAC | | |  |  |  |  |
|  |  |  |  |  |  |  |  |  |  |  | Probe | | | | GGATGGTTTAGACGACC | | |  |  |  |  |
| *nuc* | | | | SAUSA300_1222 | | | | | Thermonuclease | | | Forward primer | | | | | CCTGTACAACCATTTGGCAAAGAAGC |  |  |  |  |
|  |  |  |  |  |  |  |  |  |  |  |  | Reverse primer | | | | | GCAAGTCCCTTTTCCACTAATTCC |  |  |  |  |
|  |  |  |  |  |  |  |  |  |  |  |  | Probe | | | | | CGCTATGGTAGAACATTG |  |  |  |  |
| *SAUSA300_0693* | | | | SAUSA300_0693 | | | | | Hypothetical protein/saeP | | | Forward primer | | | | | GTGAAACTGTTGAAGGTAAAGCTG |  |  |  |  |
|  |  |  |  |  |  |  |  |  |  |  |  | Reverse primer | | | | | ACCATTGCGATTTCTTTACC |  |  |  |  |
|  |  |  |  |  |  |  |  |  |  |  |  | Probe | | | | | CAAATCATCAAAAGGTCC |  |  |  |  |
| *SAUSA300_0883* | | | | SAUSA300_0883 | | | | | Putative surface protein | | | Forward primer | | | | | CAATTGCAGTAGATGGCATTATGGC |  |  |  |  |
|  |  |  |  |  |  |  |  |  |  |  |  | Reverse primer | | | | | CTTTCCAAGTAATCGTGTAAACGGCAG |  |  |  |  |
|  |  |  |  |  |  |  |  |  |  |  |  | Probe | | | | | CCAAAAGATAGCCAATTA |  |  |  |  |
| *SAUSA300_1217* | | | | SAUSA300_1217 | | | | | ABC transporter ATP-binding protein | | | Forward primer | | | | | GGTGCTGGAAAGTCAACGTTAATTG |  |  |  |  |
|  |  |  |  |  |  |  |  |  |  |  |  | Reverse primer | | | | | GAAACATCGTTTTTTGGAACATTATACTG |  |  |  |  |
|  |  |  |  |  |  |  |  |  |  |  |  | Probe | | | | | AATTCTGGTGAGATATTTG |  |  |  |  |
| *sarA* | | | | | SAUSA300_0605 | | | | Transcriptional regulator | | | | Forward primer | | | | GAGTTGTTATCAATGGTCACTTATGCTG |  |  |  |  |
|  |  |  |  |  |  |  |  |  |  |  |  |  | Reverse primer | | | | CTTTGTTTTCGCTGATGTATGTCAATAC |  |  |  |  |
|  |  |  |  |  |  |  |  |  |  |  |  |  | Probe | | | | GAATTTTCAATTAGCTTTG |  |  |  |  |
| *sarS* | | | | | SAUSA300_0114 | | | | Transcriptional regulator | | | | Forward primer  Reverse primer  Probe | | | | GATGAGCGTAATACTTACATTTCAATATCTG  CTATCTTTTGGTATCATCTGTGATTCAC |  |  |  |  |
|  |  |  |  |  |  |  |  |  |  |  |  |  |  |  |  |  | CAGAACGTGTTACATTG |  |  |  |  |
| *sarZ* | | | | | SAUSA300_2331 | | | | MarR family regulatory protein | | | | Forward primer  Reverse primer | | | | GGTTACATTGTTTTAATGGCGATTG  CATCTTTCTCTTCACGTGTTCGAAC |  |  |  |  |
|  |  |  |  |  |  |  |  |  |  |  |  |  | Probe | | | | CTTAGATTCTGGAACACTG |  |  |  |  |
| *sasA* | | | | | SAUSA300_2589 | | | | *S. aureus* surface protein A | | | | Forward primer | | | | GCGACAAATTTACAACAAGTACAATTTGG |  |  |  |  |
|  |  |  |  |  |  |  |  |  |  |  |  |  | Reverse primer | | | | CGATTGTCACGACTTGATCAACATTTC |  |  |  |  |
|  |  |  |  |  |  |  |  |  |  |  |  |  | Probe | | | | CTGCTGTTACACAAGTG |  |  |  |  |
| *sasB* | | | | | SAUSA300_2110 | | | | *S. aureus* surface protein B | | | | Forward primer | | | | CTACTATGCAAACGAATAGTAAGCAAGG |  |  |  |  |
|  |  |  |  |  |  |  |  |  |  |  |  |  | Reverse primer | | | | GTAATTCTTGAAGCATCAGCAACTGC |  |  |  |  |
|  |  |  |  |  |  |  |  |  |  |  |  |  | Probe | | | | GAATTAGCAACTGTAAATG |  |  |  |  |
| *sasC* | | | | | SAUSA300_1702 | | | | *S. aureus* surface protein C | | | | Forward primer | | | | GGTTCAGGAGGACATCTAACTTTAAAGG |  |  |  |  |
|  |  |  |  |  |  |  |  |  |  |  |  |  | Reverse primer | | | | TGCCGCACGTCTACTTCTCTTTTTC |  |  |  |  |
|  |  |  |  |  |  |  |  |  |  |  |  |  | Probe | | | | GAGCTAGTTGCAATTGC |  |  |  |  |
| *sasD* | | | | | SAUSA300_0136 | | | | *S. aureus* surface protein D | | | | Forward primer | | | | CCTTATGGCGGAGTAGTACCACAAG |  |  |  |  |
|  |  |  |  |  |  |  |  |  |  |  |  |  | Reverse primer | | | | GCGTCGCATCATACAATTTCATATTATAG |  |  |  |  |
|  |  |  |  |  |  |  |  |  |  |  |  |  | Probe | | | | GCACAATATACTGAATTAG |  |  |  |  |
| *sasF* | | | | | SAUSA300_2581 | | | | *S. aureus* surface protein F | | | | Forward primer | | | | CATTGATTGATCAATCACAAGATAAGTCG |  |  |  |  |
|  |  |  |  |  |  |  |  |  |  |  |  |  | Reverse primer | | | | CGATTTGATAATCCTTTATTCGTCC |  |  |  |  |
|  |  |  |  |  |  |  |  |  |  |  |  |  | Probe | | | | TTACAAACGAAATTAGG |  |  |  |  |
| *sasG* | | | | | SAUSA300_2436 | | | | *S. aureus* surface protein G | | | | Forward primer | | | | CGAGAAAATACCGCAAGGTCATAAA |  |  |  |  |
|  |  |  |  |  |  |  |  |  |  |  |  |  | Reverse primer | | | | TGTCTGGATTCTTGATTCCTGGTTT |  |  |  |  |
|  |  |  |  |  |  |  |  |  |  |  |  |  | Probe | | | | CAGATCAAACGGAAAAAGTA |  |  |  |  |
| *sbi* | | | | | SAUSA300_2364 | | | | Immunoglobulin G-binding protein | | | | Forward primer | | | | GAAGAACAACGTAACCAATACATCAAAAC |  | | |  |
|  |  |  |  |  |  |  |  |  |  |  |  |  | Reverse primer | | | | GTAAAAAGCGTTTTGTTGTGCAACAC |  |  |  |  |
|  |  |  |  |  |  |  |  |  |  |  |  |  | Probe | | | | GAAGTATTCTCTGAATCAC |  |  |  |  |
| *scpA* | SAUSA300_1445 | | | | | | | | Staphopain A | | | | Forward primer | | | | CAGAGCAGTATATGCAGTACGTTCATGC | | |  |  |
|  |  |  |  |  |  |  |  |  |  |  |  |  | Reverse primer | | | | GATATTCTATTAAACGCCCAACTAAATC | | |  |  |
|  |  |  |  |  |  |  |  |  |  |  |  |  | Probe | | | | TACCACAATCAACATCAG | | |  |  |
| *sdrC* | SAUSA300_0546 | | | | | | | | Serine-aspartate repeat-containing protein | | | | Forward primer | | | | ATGAATAATAAAAAGACAGCAACAAATAGA | | |  |  |
|  |  |  |  |  |  |  |  |  |  |  |  |  | Reverse primer | | | | AGCAGTACCTACAGAATACTTTCTTATCGA | | |  |  |
|  |  |  |  |  |  |  |  |  |  |  |  |  | Probe | | | | AAAGGCATGATACCAAATCG | | |  |  |
| *spa* | SAUSA300_0113 | | | | | | | | Staphylococcal protein A | | | | Forward primer | | | | CAAACCTGGTCAAGAACTTGTTGTTG | | |  |  |
|  |  |  |  |  |  |  |  |  |  |  |  |  | Reverse primer | | | | GCTAATGATAATCCACCAAATACAGTTG | | |  |  |
|  |  |  |  |  |  |  |  |  |  |  |  |  | Probe | | | | CATGCAGATGCTAAC | | |  |  |
| *sspA* | SAUSA300_0951 | | | | | | | | Glutamyl endopeptidase/ V8 protease | | | | Forward primer | | | | CTTATATTCAAGTTGAAGCACCTACTGG | | |  | |
|  |  |  |  |  |  |  |  |  |  |  |  |  | Reverse primer | | | | CTTTTAAAGCATGAGGATCACCGTG | | |  |  |
|  |  |  |  |  |  |  |  |  |  |  |  |  | Probe | | | | GGTGTAGTTGTAGGTAAAG | | |  |  |
| sspB | SAUSA300_0950 | | | | | | | | Staphopain B | | | | Forward primer | | | | CAACAACAATTTGCTGGTTATGCTAAAG | | |  | |
|  |  |  |  |  |  |  |  |  |  |  |  |  | Reverse primer | | | | TAAACAATTTTACCGTCTTTTATAACTGG | | |  |  |
|  |  |  |  |  |  |  |  |  |  |  |  |  | Probe | | | | GTAATGCAAAAACTGG | | |  |  |

**Supplementary Table 2.** Genes that are significantly up- or down-regulated at 2, 3, 24 and 72 hours of infection, when compared with inoculated bacteria.

|  | **Downregulated** | **Upregulated** |
| --- | --- | --- |
| **2 hours of infection** | *adsA, eap* | *essB, esxB, fhuD2, esxC, esaA, isaA, esxA, isaB, ebpS, sasD, clfA, csa1A, lukE, asp23, sasF, csa3A, sasC* |
| **3 hours of infection** | *adsA* | *essB, nuc, esxB, sasA, fhuD2, esxC, isaA, isaB, ebpS, sasD, sasC, fnbA, esaA, csa3A, esxA, asp23, isdG, isdC, sasF, csa1A, lukE* |
| **24 hours of infection** | *sarS, clfB, spa* | *ebpS, esxC, esxB, esxA, isaB, essC, essB, esaA, lukS, atl, isdA, geh, isdG, mntA, csa3A, isaA, asp23, sasA, csa1A, isdC, hlgb2, SAUSA300_0883, clfA, sasF, hla, aur, sspB, sspA* |
| **72 hours of infection** | *mgrA, spa, sarS, sarZ, clfB, chp, sbi* | *esxA, ebpS, isaB, atl, isaA, esaA, esaB, esxC, SAUSA300_0883, mntA, asp23, essB, esxB, sasA, csa3A, sasF, aur, isdC, csa1A, clfA, sspB, sspA* |

**Supplementary Table 3.** Genes that were commonly up- or down-regulated at colonization (2 and 3 hours of infection) and/or infection (24 and 72 hours of infection) stages, when compared with inoculated bacteria.

|  | **Downregulated** | **Upregulated** |
| --- | --- | --- |
| **Colonization**  (2 and 3 hours of infection) | *adsA* | *essB, esxB, fhuD2, esxC, esaA, isaA, esxA, isaB, ebpS, sasD, csa1A, lukE, asp23, sasF, csa3A, sasC* |
| **Infection**  (24 and 72 hours of infection) | *sarS, clfB, spa* | *ebpS, esxC, esxB, esxA, isaB, essB, esaA, atl, mntA, csa3A, asp23, isaA, csa1A, SAUSA300_0883, sasF, aur, sspB, sspA* |
| **Colonization and Infection**  (2, 3, 24 and 72 hours of infection) | *-* | *ebpS, esxC, esxB, esxA, isaB, essB, esaA, csa3A, isaA, asp23, csa1A, sasF* |

**Supplementary References**

1. Brignoli T, Manetti AGO, Rosini R, et al. Absence of protein a expression is associated with higher capsule production in staphylococcal isolates. Front Microbiol. **2019**; 10:1–15.
